# Identification of the Components Involved in Cyclic Di-AMP Signaling in *Mycoplasma pneumoniae*

**DOI:** 10.3389/fmicb.2017.01328

**Published:** 2017-07-13

**Authors:** Cedric Blötz, Katrin Treffon, Volkhard Kaever, Frank Schwede, Elke Hammer, Jörg Stülke

**Affiliations:** ^1^Department of General Microbiology, Institute of Microbiology and Genetics, Georg-August University Göttingen Göttingen, Germany; ^2^Research Core Unit Metabolomics, Hannover Medical School Hannover, Germany; ^3^Biolog Life Science Institute Bremen, Germany; ^4^Department of Functional Genomics, Interfaculty Institute for Genetics and Functional Genomics, University Medicine Greifswald Greifswald, Germany

**Keywords:** second messenger, diadenylate cyclase, phosphodiesterase, c-di-AMP, potassium uptake, Mollicutes

## Abstract

Bacteria often use cyclic dinucleotides as second messengers for signal transduction. While the classical molecule c-di-GMP is involved in lifestyle selection, the functions of the more recently discovered signaling nucleotide cyclic di-AMP are less defined. For many Gram-positive bacteria, c-di-AMP is essential for growth suggesting its involvement in a key cellular function. We have analyzed c-di-AMP signaling in the genome-reduced pathogenic bacterium *Mycoplasma pneumoniae*. Our results demonstrate that these bacteria produce c-di-AMP, and we could identify the diadenylate cyclase CdaM (MPN244). This enzyme is the founding member of a novel family of diadenylate cyclases. Of two potential c-di-AMP degrading phosphodiesterases, only PdeM (MPN549) is active in c-di-AMP degradation, whereas NrnA (MPN140) was reported to degrade short oligoribonucleotides. As observed in other bacteria, both the c-di-AMP synthesizing and the degrading enzymes are essential for *M. pneumoniae* suggesting control of a major homeostatic process. To obtain more insights into the nature of this process, we have identified a c-di-AMP-binding protein from *M. pneumoniae*, KtrC. KtrC is the cytoplasmic regulatory subunit of the low affinity potassium transporter KtrCD. It is established that binding of c-di-AMP inhibits the KtrCD activity resulting in a limitation of potassium uptake. Our results suggest that the control of potassium homeostasis is the essential function of c-di-AMP in *M. pneumoniae*.

## Introduction

To respond to changes in their environment, bacteria have evolved a large set of signal detection and transduction systems. Such signals can be directly sensed by proteins, they may feed into complex networks that control gene expression via transcription factors, or they are first converted to so-called second messengers which in turn provoke a response. Many bacteria use dedicated nucleotides as second messengers, among them cyclic AMP, (p)ppGpp and the cyclic dinucleotides c-di-AMP and c-di-GMP (Gomelsky, 2011).

cAMP is the paradigmatic second messenger, and this nucleotide is involved in coordinating carbon and nitrogen metabolism and in carbon catabolite repression in *Escherichia coli* and related bacteria (Görke and Stülke, 2008; You et al., 2013). (p)ppGpp is formed upon starvation and triggers a reduction of cellular house-keeping activities (Steinchen and Bange, 2016). Cyclic di-GMP is in many bacteria involved in the choice between sessile and motile lifestyles (Hengge, 2009). While these second messengers have been intensively studied in a large number of different bacteria, cyclic di-AMP has been discovered only less than a decade ago (Witte et al., 2008). This nucleotide was found in the crystal structure of the so-called DNA integrity scanning protein DisA, which exhibits diadenylate cyclase activity (Witte et al., 2008). C-di-AMP is formed in a variety of both Gram-positive and Gram-negative bacteria with the notable exception of the gamma-proteobacteria including the enterobacteria. Several studies have revealed that this second messenger is essential for many Gram-positive bacteria with a low genomic GC content (the Firmicutes). This has been shown for *Bacillus subtilis*, *Staphylococcus aureus*, *Listeria monocytogenes*, and many other species (Luo and Helmann, 2012; Corrigan and Gründling, 2013; Commichau et al., 2015). Moreover, the accumulation of c-di-AMP has also been shown to cause severe problems for the cells (Mehne et al., 2013; Gundlach et al., 2015b). To control the intracellular levels of c-di-AMP, the bacteria that produce cyclic di-AMP do also possess phosphodiesterases to degrade this molecule (Rao et al., 2010; Corrigan et al., 2011; Commichau et al., 2015; Huynh and Woodward, 2016).

**FIGURE 1:**
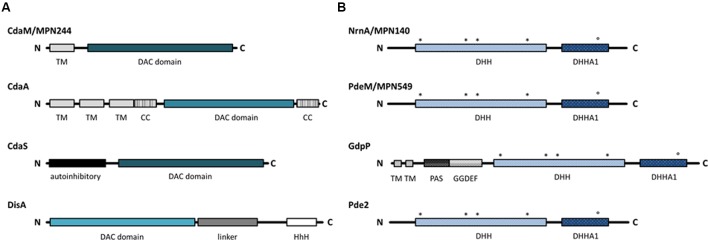
Domain architecture of c-di-AMP related enzymes. Black lines indicate protein sequences and the boxes represent domains. **(A)** The four classes of c-di-AMP producing DACs. Intensity of the blue color indicates differences in the DAC domain. **(B)** Phosphodiesterases specific for c-di-AMP degradation. Conserved residues in the DHH and DHHA1 motifs are indicated with asterisks and circle, respectively. DAC, di-adenylate cyclase; TM, transmembrane domain; CC, coiled-coil domain; HhH, Helix-hairpin-Helix; PAS, Per-Arnt-Sim sensor domain; DHH/DHHA/GGDEF, Asp-His-His/ Asp-His-His-Ala/ Gly-Gly-Asp-Glu-Phe.

All known diadenylate cyclases share a conserved domain, the so-called DAC domain (Römling, 2008; Commichau et al., 2015; Rosenberg et al., 2015). However, the DAC domain can be found in different arrangements with other domains. Based on the domain organization, three classes of diadenylate cyclases have been studied so far (see **Figure 1**). The proteins of the CdaA class are membrane proteins with three transmembrane domains at the N-terminus (Gundlach et al., 2015b). This class of diadenylate cyclases is the most widespread, and it is ubiquitous in most Firmicutes. CdaS is a cyclase that is only found in *B. subtilis* and closely related spore formers. This cyclase consists of an N-terminal autoinhibitory domain and the DAC domain (Mehne et al., 2014). Finally, the enzymes of the DisA class are found in spore-forming Firmicutes (*Bacillus* spp., *Clostridium* spp.) and in the Actinobacteria. These octameric enzymes have their DAC domain at the N-terminus, and bind DNA via a C-terminal helix-hairpin-helix domain (Witte et al., 2008; Commichau et al., 2015). While most bacterial species contain one diadenylate cyclase, *B. subtilis* encodes three enzymes, one of each class (Mehne et al., 2013). Two principal classes of c-di-AMP-degrading phosphodiesterases have been described: The proteins of the first class possess a domain called DHH-DHHA1 with a catalytic Asp-His-His motif. The DHH-DHHA1 domain can either be part of a larger protein as in *B. subtilis* GdpP or exert the enzymatic activity without any additional domains as described for *Streptococcus pneumoniae* Pde2 (see **Figure 1**, Rao et al., 2010; Bai et al., 2013). The enzymes of the second class possess a so-called HD domain with a His-Asp catalytic motif (Huynh et al., 2015).

While the functions of cAMP, (p)ppGpp, and c-di-GMP are well understood, this is not the case for c-di-AMP. Earlier studies have implicated c-di-AMP in the control of cell wall homeostasis (Corrigan et al., 2011; Luo and Helmann, 2012; Mehne et al., 2013). Moreover, c-di-AMP formation by DisA was suggested to be important for integrity control, repair and recombination of DNA (Oppenheimer-Shaanan et al., 2011; Campos et al., 2014). The isolation of c-di-AMP binding proteins has identified the small signal transduction protein DarA, the pyruvate carboxylase, subunits of potassium transporters, and ATP-binding subunits of osmoprotectant ABC transporters (Corrigan et al., 2013; Sureka et al., 2014; Gundlach et al., 2015a; Huynh et al., 2016; Schuster et al., 2016). Moreover, c-di-AMP binds and inhibits the KdpD sensor kinase that controls the expression of a high-affinity potassium transporter in *S. aureus* (Moscoso et al., 2016). Finally, c-di-AMP binds an RNA molecule, i.e., the riboswitch that controls the expression of the potassium transporters KimA and KtrAB in *B. subtilis* (Nelson et al., 2013; Gundlach et al., 2017). Binding to both KtrA and the riboswitch controlling its expression makes c-di-AMP the only second messenger that controls a biological process (potassium uptake) by binding both to a protein and to the corresponding mRNA molecule (Commichau et al., 2015). Among all identified targets of c-di-AMP, not a single one is essential. Only recently, c-di-AMP essentiality could be traced back to the control of potassium homeostasis in *B. subtilis* (Gundlach et al., 2017).

We are interested in signal transduction in the strongly genome-reduced pathogenic bacterium *Mycoplasma pneumoniae*. This bacterium encodes only 694 proteins (Lluch-Senar et al., 2015), reflecting its adaptation to rather constant environmental conditions in the natural habitat, human lung epithelia. Accordingly, *M. pneumoniae* possesses only three putative transcription factors to control gene expression. However, the mechanisms of transcriptional regulation in *M. pneumoniae* are still poorly understood (Güell et al., 2009). In addition, the second messenger ppGpp is likely formed by *M. pneumoniae* as deduced from the presence of a ppGpp synthetase-encoding gene (Eilers, 2010).

In this study, we have analyzed the presence of components involved in c-di-AMP signaling in *M. pneumoniae*. We demonstrate that this second messenger is formed by this bacterium, and have identified the enzymes responsible for its synthesis and degradation. Moreover, we have discovered that c-di-AMP binds to the *M. pneumoniae* KtrC protein, indicating a function in the control of potassium uptake.

## Materials and Methods

### Bacterial Strains and Growth Conditions

*Mycoplasma pneumoniae* was handled under L2 laboratory safety conditions. The *M. pneumoniae* strain used in this study was *M. pneumoniae* M129 (ATCC 29342). *M. pneumoniae* was grown at 37°C in 175 cm^2^ tissue culture flasks containing 100 ml of modified Hayflick medium as described previously (Halbedel et al., 2004). Carbon sources were added to a final concentration of 1% (w/v). *Escherichia coli* XL1blue and BL21(DE3)/pLysS (Sambrook et al., 1989) were used as host for cloning and recombinant protein expression, respectively. *E. coli* strains were cultivated in Luria Bertani broth (Sambrook et al., 1989) containing ampicillin 100 μg ml^-1^. *Bacillus subtilis* 168 was used to extract chromosomal DNA.

### DNA Manipulation and Plasmid Construction

Chromosomal DNA from *B. subtilis* and *M. pneumoniae* was isolated using the Blood and Tissue Kit according to the manufacturer’s instructions (Qiagen, Hilden, Germany). Transformation of *E. coli* and plasmid DNA extraction was performed using standard procedures (Sambrook et al., 1989).

Plasmids for the overexpression and purification of the enzymes potentially involved in c-di-AMP metabolism from *M. pneumoniae* were constructed as follows. The coding sequence of each gene was amplified by PCR with gene specific primers (listed in Supplementary Table S1) using chromosomal DNA of *M. pneumoniae* M129 as the template. As the *M. pneumoniae* genes contain TGA codons that code for tryptophan in *M. pneumoniae* but for an opal stop codon in *E. coli*, the PCR fragments were used as templates for mutagenesis by the multiple mutation reaction (Hames et al., 2005) using the amplification primers and 5′-phosphorylated mutagenic primers (listed in Supplementary Table S1) to introduce TGA to TGG transitions. The cytoplasmic portion of the *B. subtilis gdpP* gene (GdpP_84-659_) was amplified from *B. subtilis* chromosomal DNA. The PCR products of genes potentially encoding phosphodiesterases were digested with BamHI and NdeI and cloned into the expression vector pGP574 (Schilling et al., 2006). This plasmid allows the expression of enzymes carrying a C-terminal Strep-tag. The resulting plasmids are pGP2717 (*mpn140*), pGP2718 (*mpn549*), and pGP2720 (*gdpP*). The PCR product for the potential diadenylate cyclase-encoding gene *mpn244* was cleaved with NdeI and BamHI and cloned into the vector pET3c (Novagene, Darmstadt, Germany). The resulting plasmid was pGP2036. All plasmid inserts were verified by DNA sequencing.

### Protein Overexpression and Purification

The Strep-tagged proteins were overexpressed in *E. coli* BL21(DE3). Expression (1 l culture, 37°C, 200 rpm, baffled flasks) was induced by the addition of 1 mM IPTG to exponentially growing cultures (OD_600_ of 0.6 to 0.8). After expression for 3 hours, the cells were pelleted at 4°C for 20 min at 4,000 rpm and washed once with 20 ml PDE buffer (100 mM Tris-HCl pH8.3, 150 mM NaCl, 1 mM EDTA, 5 % glycerol). Cells were lysed using a French press (18,000 p.s.i., 138,000 kPa, three passes, SLM Aminco, United States). After lysis, the crude extracts were centrifuged at 35 000 r.p.m for 30 min. The crude extract was then passed over a StrepTactin column (IBA, Göttingen, Germany). The recombinant proteins were eluted with desthiobiotin (IBA, final concentration 2.5 mM). After elution, the fractions were tested for the desired protein using 12% SDS-PAGE. Only fractions which contained the desired protein in apparent homogeneity (content of the specific protein >95%) were used for further purification by running the proteins over a 10 ml HiTrap Heparin HP column (GE Healthcare) using the ÄKTA prime plus system (GE Healthcare, flow rate 2 ml/min). With 1 M NaCl in the PDE buffer, proteins were eluted from the column. Fractions containing pure protein were concentrated and dialyzed to 1.5 ml without NaCl in PDE buffer using Vivaspin^®^Turbo15 ultrafiltration spin columns (MW 5,000 Da; Sartorius, Göttingen, Germany). Protein concentrations were determined using the Bio-Rad dye-binding assay where bovine serum albumin served as the standard. Aliquots of the proteins were frozen in liquid nitrogen and stored at –80°C prior to further experiments.

### Enzyme Assays

The assays for phosphatase and phosphodiesterase activities of Strep-tagged proteins were performed as described previously (Diethmaier et al., 2014). Phosphatase activity against *para*-nitrophenol phosphate (*p*NPP) was assayed in a buffer containing 100 mM Tris-HCl pH8.3, 10 mM NaCl, 0.1 mM MnCl_2_, 25 mM *p*NPP and different amounts of purified protein in a total volume of 100 μl. Phosphodiesterase activity against bis-*p*NPP was assayed in a buffer containing 100 mM Tris-HCl pH8.3, 10 mM NaCl, 0.1 mM MnCl_2_, or MgCl_2_, 1–7.5 mM bis-*p*NPP and purified protein in a total volume of 100 μl. The reactions were initiated by the addition of the protein, and the reaction mixture was incubated at 37°C for 4 h. Relative substrate cleavage was quantified by measuring the OD_410_ using a microplate reader (EPOCH| 2, BioTek, Winooski, United States).

Phosphodiesterase activity with cyclic dinucleotides was measured using a quantitative assay based on the interaction of c-di-AMP with the fluorescent dye coralyne (Zhou et al., 2014). Briefly, reaction mixtures (150 μl) consisted of 100 mM Tris-HCl pH8.3, 10 mM NaCl, 0.1 mM MnCl_2_, and 100 μM c-di-AMP. The reaction was initiated by addition 100 nM enzyme (for MPN549 additionally 50 and 10 nM), and the reaction mixture was incubated at 37°C for 4 h. At different time points the reactions were stopped by addition of 1 μl EDTA (0.5 M), shock-freezing and subsequent boiling for 10 min. To quantify the remaining c-di-AMP, the samples were spun to remove protein precipitate. KBr and coralyne were added to the sample to final concentrations of 250 mM and 10 μM, respectively. After 20 min of incubation at 37°C in the dark, relative c-di-AMP cleavage was determined as described (Zhou et al., 2014) by measuring the fluorescence emission at 475 nm using a microplate reader (SYNERGY Mx, BioTek, Winooski, United States).

In all kinetic experiments, *K*_m_ and *V*_max_ were determined by nonlinear curve fitting from Lineweaver-Burk plots.

### c-di-AMP Extraction

The concentrations of c-di-AMP in *E. coli* and in *M. pneumoniae* cells were determined by a liquid chromatography tandem MS (LC-MS/MS) method, essentially as described previously (Mehne et al., 2013; Gundlach et al., 2015b). Briefly, *E. coli* or *M. pneumoniae* cells were grown in LB (20 ml) or MP (100 ml) medium, respectively. For *E. coli* two additional aliquots (1 ml each) of each sample were harvested for total protein determination. For *M. pneumoniae*, the wet weight of each sample was determined. The pellets were resuspended in 300 μl extraction mixture (acetonitrile/methanol/water 40/40/20 v/v/v) and shock-frozen in liquid nitrogen, followed by boiling for 10 min. The boiled samples were centrifuged for 10 min at 4°C and 20,800 *g*. The supernatants were stored on ice, and the remaining pellets were used for two more extraction steps with 200 μl extraction mixture. For this purpose, the samples were mixed, incubated on ice for 15 min, and centrifuged again. The obtained supernatants were pooled. The samples were incubated at –20°C over night, and centrifuged again (20 min, 4°C, 20,800 *g*). Then, the supernatant was transferred to a fresh reaction tube, and dried in a speed vac at 50°C for 2 h and resuspended in 200 μl of water. After repeated centrifugation and addition of the internal standard ([^13^C,^15^N]c-di-AMP), part of the extract was analyzed by LC-MS/MS.

### Quantification of c-di-AMP by MS/MS

Chromatographic separation was performed on a series 200 high-pressure liquid chromatography (HPLC) system (PerkinElmer Life Sciences) as described previously (Mehne et al., 2013). The analyte detection was performed on an PI4000 triple-quadrupole mass spectrometer equipped with an electrospray ionization source (AB Sciex), using selected reaction monitoring (SRM) analysis in positive ionization mode. The SRM transitions labeled “quantifier” were used to quantify the compound of interest, whereas “identifier” SRM transitions were monitored as confirmatory signals. The quantifier SRM transitions were most intense and were therefore used for quantification.

### Pull Down of c-di-AMP Binding Proteins Via c-di-AMP Coupled Agarose

To isolate c-di-AMP-binding proteins from *M. pneumoniae*, we made use of immobilized c-di-AMP (2′-*O*-(6-aminohexylcarbamoyl)-cyclic diadenosine monophosphate on agarose (2′-AHC-c-di-AMP-agarose; BIOLOG, Bremen, Germany). We prepared *M. pneumoniae* crude cell extracts from 150 mg cells per assay. The cells were lysed in pulldown buffer (100 mM Tris-HCl pH7.5, 100 mM KCl, 150 mM NaCl, 5 mM MgCl_2_, 0.5 mM DTT, 0.1% (v/v) Tween-20, and 1x cOmplete^TM^ protease inhibitor (Roche Diagnostics, Mannheim, Germany)). Cells were lysed in a tissue lyser with 0.1 mm glass beads (2 × 2.5 min, 30 Hz, cooled block) followed by ultracentrifugation for 30 min at 35,000 rpm and 4°C. To prepare the matrix, 500 μl 2′-AHC-c-di-AMP agarose or ethanolamine-coupled agarose (negative control) were equilibrated three times with 1 ml of pulldown buffer. After equilibration, the cell lysate and the agarose matrix were incubated rotating over night at 4°C. The supernatant was discarded and the matrix was washed three times (1 ml 1x PBS; 137 mM NaCl, 2.7 mM KCl, 10 mM Na_2_HPO_4_, 1.76 mM KH_2_PO_4_, pH7.4). Elution of binding proteins was performed with 200 μl of 1x PBS, pH7.4 containing 1 mM c-di-AMP and 15 min incubation at RT and 1,400 rpm. The eluted proteins were concentrated using acetone precipitation (1:1 sample:acetone) over night at –20°C. The precipitate was centrifuged 1 h at 14,800 rpm and 4°C. The remaining supernatant was discarded and the sample dried for 10 min at RT. The remaining pellet was solved in 40 μl 1x PBS and analyzed by SDS-PAGE. Proteins of interest were identified by mass spectrometry as described previously (Meyer et al., 2011). Briefly, after destaining of gel slices, proteins underwent in-gel digestion with trypsin (10 ng/ μl trypsin in 20 mM ammonium bicarbonate). Peptides were separated by C18 reverse phase liquid chromatography (nanoAcquity UPLC system, 10 cm, Waters, Manchester, United Kingdom) in a linear gradient of 0.1 % acetic acid in acetonitrile from 5 % up to 25 % within 65 min (flow rate: 400 nl/min). MS analysis was performed on a LTQ-Orbitrap Velos hybrid mass spectrometer (Thermo Electron, Bremen, Germany) operated in data-dependent MS/MS mode. Proteins were identified by searching all MS/MS spectra against a *M. pneumoniae* M129 protein database (687 entries, extracted from Uniprot rel. 05-2014) using Sequest algorithm on a SorcererTM software platform. Initial mass tolerance for peptide identification on MS and MS/MS peaks were 10 ppm and 1 Da, respectively. Up to two missed tryptic cleavages were allowed. Methionine oxidation (+15.99492 Da) and propionamide modification on cysteine (+71.037109 Da) were set as variable modifications. Protein identification results were evaluated by determination of probability for peptide and protein assignments provided by PeptideProphet and ProteinProphet (ISI Seattle, WA, United States) incorporated in the Scaffold software package rel. 4.3.2 (Proteome Software, Portland, OR, United States). Proteins were identified by at least two peptides with peptide probability >90% reflecting protein probability of >99%.

### Assay for *M. pneumoniae* Mutants

For the isolation of *M. pneumoniae* mutants, we used an ordered collection of strains carrying insertions of transposon Tn4001 (Halbedel et al., 2006). The presence of the desired mutant was assayed by a PCR screen using one primer that hybridizes to the transposon (directed outward), and a second primer specific for the gene of interest (see Supplementary Table S1).

## Results

### *M. pneumoniae* Proteins Potentially Involved in c-di-AMP Metabolism

All Firmicutes studied so far produce c-di-AMP, and in most of them, this second messenger is essential for the growth of these bacteria (Commichau et al., 2015). However, the presence of this signaling nucleotide has never been analyzed in representatives of the Mollicutes. To address this question for *M. pneumoniae*, we cultivated cells, extracted nucleotides, and analyzed these extracts for the presence of c-di-AMP. The total amount of c-di-AMP (nM) extracted from the pellet was divided by the total cell volume (mg) to yield the c-di-AMP concentration per mg cells. In three independent biological experiments (with three technical replicates each), we determined concentrations of 489 ± 107 nM, 958 ± 153 nM, and 859 ± 81 nM. Such differences among biological replicates are not unusual when assaying c-di-AMP concentrations (Gundlach et al., 2015b). Importantly, our results demonstrate that *M. pneumoniae* does possess c-di-AMP, and imply the existence of enzymes that synthesize and degrade this second messenger.

To identify candidate genes involved in *M. pneumoniae* c-di-AMP metabolism, we analyzed the genome for genes encoding proteins possessing a c-di-AMP synthesizing DAC domain as well as for proteins containing c-di-AMP degrading DHH-DHHA1 and HD domains. A single protein containing a DAC domain was found. The DAC domain of this protein, MPN244, is most similar to the domain of the *B. subtilis* sporulation-specific cyclase CdaS. However, the N-terminal domain of this protein is not similar to any domains found in other characterized diadenylate cyclases. According to the UniProt database, the amino acids 6 to 26 form a transmembrane helix. Thus, if MPN244 is endowed with diadenylate cyclase activity, this protein would represent a novel class of diadenylate cyclases. A comparison of the domain arrangement of the different classes of diadenylate cyclases is shown in **Figure 1A**.

An analysis of potential c-di-AMP degrading phosphodiesterases in *M. pneumoniae* failed to identify a protein containing a HD domain similar to that of the *B. subtilis* phosphodiesterase PgpH (Huynh et al., 2015). In contrast, the search for proteins containing a DHH–DHHA1 domain resulted in the identification of two proteins, MPN140 and MPN549. These proteins have been annotated as nano-RNase (NrnA) and probable single-stranded-DNA-specific exonuclease (RecJ) (Postic et al., 2012; Wodke et al., 2015), respectively. Two classes of c-di-AMP specific DHH-DHHA1 phosphodiesterases have been identified: a class of large membrane-bound proteins, as exemplified by *B. subtilis* GdpP. This protein contains an N-terminal transmembrane domain, a PAS domain, and a degenerate GGDEF domain possibly involved in c-di-GMP sensing in addition to the DHH-DHHA1 domain. In contrast, the second class, represented by Pde2 of *Streptococcus pneumoniae*, consists only of the DHH-DHHA1 domain (Bai et al., 2013). Both potential phosphodiesterases of *M. pneumoniae* are similar to Pde2 rather than to GdpP (see **Figure 1B** for domain organization).

### CdaM (MPN244) Is a Diadenylate Cyclase

In order to test whether MPN244 does indeed exhibit diadenylate cyclase activity, we took advantage of the inability of *E. coli* to produce c-di-AMP (Corrigan et al., 2011). The *mpn244* gene was cloned into the expression vector pET3c, and the amounts of c-di-AMP produced in *E. coli* BL21(DE3) carrying the corresponding plasmid pGP2036, were compared to those of the same strain carrying the empty vector pET3c. As observed previously (Mehne et al., 2013), no c-di-AMP was present in the strain with the empty vector. In contrast, the expression of MPN244 resulted in the detection of 47.3 ± 11.8 pmol of c-di-AMP/mg of protein. This result provides unequivocal evidence for the diadenylate cyclase activity of MPN244, and we renamed the protein accordingly CdaM (c-di-AMP synthase of *Mycoplasma*), and the gene *cdaM*.

As c-di-AMP is essential in many Gram-positive bacteria, we wondered whether this is also the case in *M. pneumoniae*. To address this question, we attempted the isolation of a *cdaM* mutant from our transposon mutant library. This library contains 2,976 independent individual transposon mutants, and the probability to find mutants for any non-essential gene is as high as 99.999% (Halbedel et al., 2006). In agreement with previous results (Lluch-Senar et al., 2015), no mutant could be isolated suggesting that the *cdaM* gene and thus c-di-AMP is essential in *M. pneumoniae*.

### Phosphodiesterases Involved in the Degradation of c-di-AMP

**FIGURE 2:**
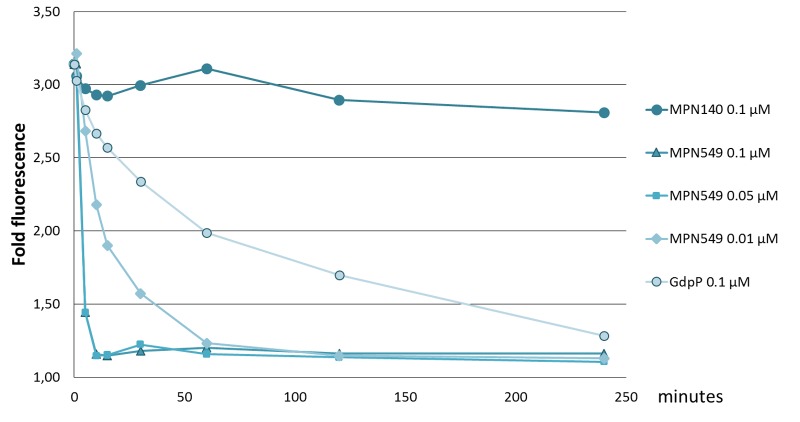
Specific activity of phosphodiesterases toward c-di-AMP. Phosphodiesterases (MPN140, MPN549, GdpP: 100 nM, in addition 50 and 10 nM for MPN549) were added to 100 μM c-di-AMP in 100 mM Tris-HCl pH8.3, 10 mM NaCl, 0.1 mM MnCl_2_ at 37°C. Reactions were stopped at 0, 5, 10, 15, 30, 60, 120, and 240 min. Upon addition of KBr and coralyne and excitation at 420 nm, fluorescence at 475 nm was measured. The ratio of c-di-AMP-mediated emission vs. a standard without c-di-AMP is shown. Results from a typical experiment are shown. The replicates showed similar results.

As mentioned above, two potential c-di-AMP degrading phosphodiesterases, MPN140 and MPN549, are encoded in the genome of *M. pneumoniae*. To assess their activity, we expressed and purified these enzymes carrying a C-terminal Strep-tag. Initially, we determined the phosphatase and phosphodiesterase activities of both enzymes. To determine phosphatase activities, we used *p*NPP, a synthetic phosphatase substrate, and the *M. pneumoniae* protein phosphatase PrpC (Halbedel et al., 2006; Schmidl et al., 2010) as a positive control. While PrpC exhibited a strong phosphatase activity, only negligible activity was detected for MPN140 and MPN549 (data not shown). Phosphodiesterase activity was assayed against bis-*p*NPP as the substrate, and the *B. subtilis* phosphodiesterase GdpP served as the control. As shown in **Table 1**, all three enzymes exhibited phosphodiesterase activity against this non-specific substrate. Thus, MPN140 and MPN549 are specifically active against phosphodiesters. In order to explore the activity of these enzymes against c-di-AMP, we determined c-di-AMP degradation using binding of c-di-AMP to the fluorescence dye coralyne as an assay. As shown in **Figure 2**, *B. subtilis* GdpP was capable of degrading the nucleotide. For MPN140, no activity against c-di-AMP was observed. In contrast, MPN549 exhibited high activity against c-di-AMP. As little as 10% of the enzyme amount used in the assay with GdpP degraded the nucleotide more rapidly than GdpP. To conclude, our results clearly demonstrate that MPN140 is a phosphodiesterase, but with a substrate distinct from c-di-AMP whereas MPN549 is the c-di-AMP-degrading phosphodiesterase in *M. pneumoniae*. Thus, MPN549 was renamed PdeM (phosphodiesterase of *M. pneumoniae*), and the gene accordingly *pdeM*.

**Table 1:** Kinetic parameters for phosphodiesterase activity on bis-pNPP.

Protein	*K*_m_ [mM]		*V*_max_ [μmol min^-1^ mmol^-1^]	
	+ MnCl_2_	+ MgCl_2_	+ MnCl_2_	+ MgCl_2_
MPN140	1.60	2.90	0.22	0.19
MPN549	0.60	0.55	0.04	0.04
GdpP	1.45	1.64	0.02	0.02

In *B. subtilis*, c-di-AMP is not only essential but also toxic in the absence of the phosphodiesterases that degrade the nucleotide (Gundlach et al., 2015b). Thus, we tested whether a *pdeM* mutant could be isolated from our transposon mutant collection. As observed in a global transposon mutant study (Lluch-Senar et al., 2015), no mutant could be isolated indicating that the *pdeM* gene is essential in *M. pneumoniae*.

### Identification of the c-di-AMP Target Protein KtrC (MPN461)

**FIGURE 3:**
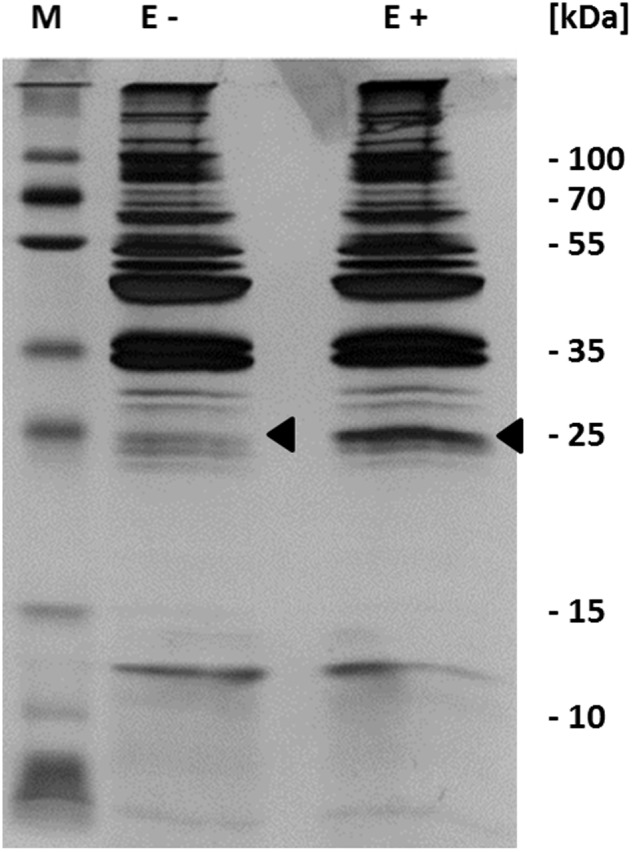
Identification of c-di-AMP binding proteins. Detection of *in vitro* c-di-AMP binding was performed with c-di-AMP immobilized to an agarose matrix. This was incubated with cell extract from *M. pneumoniae*. The c-di-AMP bound proteins were eluted with free c-di-AMP and analyzed by SDS-polyacrylamide gel electrophoresis and visualized by silver staining. Proteins were identified by mass spectrometry. Arrows show mass spectrometry analyzed bands. E, elution fraction; +, agarose coupled with c-di-AMP; -, agarose coupled with ethanolamine.

**FIGURE 4:**
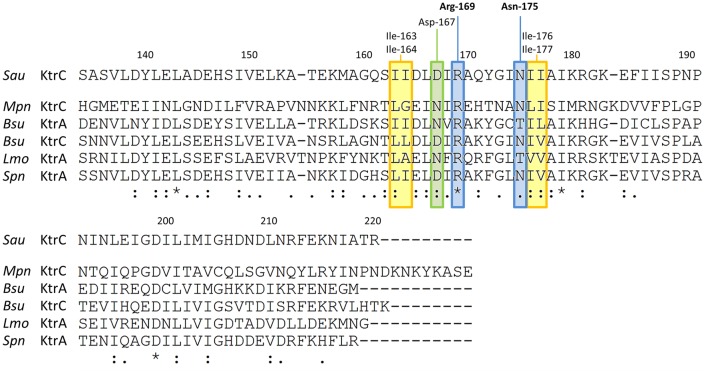
Alignment of the c-di-AMP-binding regions in RCK_C domains of potassium uptake proteins. The C-terminal parts of different KtrC/ KtrA proteins were aligned to the experimentally identified c-di-AMP binding site of *Staphylococcus aureus* (*Sau*) KtrC (Kim et al., 2015). Residues responsible for c-di-AMP binding are highlighted: Arg-169 and Asn-175 (blue), which directly interact with c-di-AMP are conserved in *M. pneumoniae* KtrC; Asp-167 (green), which also forms a direct hydrogen bond with c-di-AMP is replaced by Asn which is also known to form hydrogen bonds, Ile-163/Ile-164 and Ile-176/Ile-177 (in yellow), which form the hydrophobic patch are well conserved in bacteria. *Mpn Mycoplasma pneumoniae, Bsu Bacillus subtilis, Spn Streptococcus pneumoniae, Lmo Listeria monocytogenes*. ^⋆^ identical amino acids, **:** similar amino acids.

Our results indicate that c-di-AMP is synthesized in *M. pneumoniae*, and that this second messenger is essential for the growth of the bacteria. This suggests an important physiological function of c-di-AMP. To identify this function, we decided to isolate c-di-AMP-binding proteins, which are excellent candidates for c-di-AMP-mediated regulation. For this purpose, we used c-di-AMP immobilized via a linker to agarose and incubated it with a protein extract of *M. pneumoniae*. As a control, we used agarose containing ethanolamine immobilized via the same linker. After extensive washing (see Materials and Methods), c-di-AMP-binding proteins were eluted with free c-di-AMP. The proteins were analyzed by SDS-PAGE (see **Figure 3**), and a band exhibiting increased intensity in the binding assay identified by mass spectrometry as MPN461. This protein is similar to the *B. subtilis* low affinity potassium transporter regulatory subunit KtrC (Holtmann et al., 2003). The *mpn461* gene forms a gene cluster with the upstream gene *mpn460*, which encodes a protein most similar to the integral membrane subunit KtrD of the KtrC-KtrD potassium channel. An alignment of the c-di-AMP-binding region of *S. aureus* (Kim et al., 2015) with the relevant residues of *M. pneumoniae* and related proteins from other bacteria indicates that the amino acids interacting with c-di-AMP are conserved in the *M. pneumoniae* protein (see **Figure 4**). Taken together, these results suggest that c-di-AMP controls potassium transport in *M. pneumoniae*.

## Discussion

In this work, we provide the first analysis of c-di-AMP signaling in the genome-reduced bacterium *M. pneumoniae*. We have identified the diadenylate cyclase CdaM as well as the c-di-AMP degrading phosphodiesterase PdeM. Moreover, we have demonstrated binding of c-di-AMP to KtrC, the regulatory subunit of a potassium transporter.

So far, three distinct classes of diadenylate cyclases have been characterized. The *M. pneumoniae* CdaM protein is the prototype of a fourth class that is specific to the genome-reduced members of the genus *Mycoplasma*. The domains present in the diadenylate cyclases in addition to the enzymatically active DAC domain are thought to be required for signal recognition by the cyclases and thereby to control the activity of the enzymes (Mehne et al., 2014; Commichau et al., 2015). Thus, it is tempting to speculate that the single transmembrane domain associated with CdaM reflects the specific lifestyle of the highly host-dependent and host-adapted *Mycoplasma* cells. Taking into account the highly peculiar physiology and cell biology of the *Mycoplasmas*, it seems likely that the organization of CdaM is an adaptation to this particular biology.

The phosphodiesterase PdeM consists of a single DHH-DHHA1 domain. This type of c-di-AMP-specific phosphodiesterases has been found in other pathogenic bacteria, including *S. pneumoniae*, *Borrelia burgdorferi*, and *Mycobacterium tuberculosis*. While *S. pneumoniae* encodes both a classical phosphodiesterase (similar to GdpP, see **Figure 1**), the latter bacteria possess only the one-domain proteins, as observed in *M. pneumoniae* (Bai et al., 2013; Manikandan et al., 2014; Ye et al., 2014). In addition to PdeM, *M. pneumoniae* possesses a very similar protein, NrnA (MPN140). The two proteins share 29% identity, which is indicative of functional specialization. Indeed, while both proteins exhibit phosphodiesterase activity toward a generic substrate, only PdeM is able to degrade c-di-AMP. This is in good agreement with the reported activity of NrnA toward short oligoribonucleotides (Postic et al., 2012). Based on the results obtained with other cytoplasmic c-di-AMP specific phosphodiesterases (Bai et al., 2013; Manikandan et al., 2014; Ye et al., 2014), it is tempting to speculate that PdeM may degrade c-di-AMP to pApA, which is in turn degraded to AMP by the activity of NrnA. It is interesting to note that the presence of closely related proteins of which only one has the suspected biochemical activity is not unprecedented in *M. pneumoniae*: of the two potential glycerophosphate phosphodiesterases, only one protein (GlpQ) has the corresponding enzymatic activity, whereas no function has so far been identified for the second protein (MPN566) (Schmidl et al., 2011).

While the reason for c-di-AMP essentiality has long been enigmatic, recent research has suggested that the control of potassium homeostasis is the essential function (Gundlach et al., 2017). Three lines of evidence support this idea: (i) c-di-AMP controls potassium uptake in *B. subtilis*, *L. monocytogenes*, and *S. aureus* both at the level of transporter activity and transporter expression, and it does so by binding very different classes of proteins and even RNA. (ii) In *B. subtilis*, c-di-AMP becomes non-essential if the bacteria are cultivated at low potassium concentrations. (iii) The control of potassium uptake is the common denominator of c-di-AMP signaling in all organisms that have been studied in this respect. The demonstration that even in the genome-reduced bacterium *M. pneumoniae* c-di-AMP binds to the regulatory subunit of the KtrCD potassium transporter is in strong support for a general involvement of c-di-AMP in the control of potassium homeostasis. Moreover, the essentiality of both the diadenylate cyclase CdaM and the phosphodiesterase PdeM indicates that essentiality is not derived from the control of a single protein, but that it is rather related to a homeostatic process that is essential for the cell but that needs to be limited. This reflects the nature of potassium which is essential for any living cell but which becomes toxic if it accumulates in the cell to non-physiological concentrations (Nissen et al., 2000; Gundlach et al., 2017).

The essentiality of c-di-AMP synthesizing and degrading enzymes and the absence of c-di-AMP and c-di-AMP metabolizing enzymes from human cells makes these proteins excellent candidates for the development of novel antimicrobial drugs. Our work clearly suggests an important function for cyclic di-AMP in *M. pneumoniae*, but further work will be required to understand the molecular mechanisms by which the activity of CdaM is controlled as well as the regulation of KtrC activity by c-di-AMP.

## Author Contributions

CB, KT, and JS designed the study. CB and KT performed the experiments. VK quantified the nucleotides. FS prepared the c-di-AMP-coupled agarose. EH identified the proteins. CB and JS wrote the paper.

## Conflict of Interest Statement

The authors declare that the research was conducted in the absence of any commercial or financial relationships that could be construed as a potential conflict of interest.
