# Supplementary material for: Identification of the Components Involved in Cyclic Di-AMP Signaling in Mycoplasma pneumoniae
Source: Front Microbiol. 2017 Jul 13;8:1328. doi: 10.3389/fmicb.2017.01328 (PMC5508000; doi:10.3389/fmicb.2017.01328)
Supplement: Supplementary file 1 [file Table_1.DOCX]

**TableS1:** Primers used in this study.

| **Primer** | **Sequence** | **Restriction sites** |
| --- | --- | --- |
| **Construction of *mpn244, mpn140, mpn549* and *gdpP* overexpression plasmids** | |  |
| ***mpn244*-plasmid (pGP2036)** | |  |
| AS95 | 5‘‑AAACATATGATGATGACAGTGGAAGTCTTTTCG | NdeI |
| AS96 | 5‘‑TTTGGATCCTTAACCCGGCTTTACCGTCAG | BamHI |
| ***mpn140*-plasmid (pGP2717)** | |  |
| KT21 | 5‘‑AAACATATGAATAGCCAAGTACACCGCAAG | NdeI |
| KT22 | 5‘‑TTTGGATCCTACAGCAATTTGCTTTTGGCAATCTTG | BamHI |
| KT23 | 5‘‑p-AACTTTCCC**TGG**TTGGAGATGGTTTTCAC |  |
| KT24 | 5‘‑p-TAATACATCA**TGG**AGAACACTATATCTAGGATCTATG |  |
| ***mpn549*-plasmid (pGP2718)** | |  |
| KT25 | 5‘‑AAACATATGATTAACATCGATCCCCATTTTATTC | NdeI |
| KT26 | 5‘‑TTTGGATCCGCTGTTGACATGCTTTTGTTTGG | BamHI |
| KT27 | 5‘‑p-AAGACATTAAGAAG**TGG**ATTGGTTCCATTCGTTC |  |
| ***gdpP*-plasmid (pGP2720)** | |  |
| KT30 | 5‘‑AAACATATGCCGATTGGAATCATGCTTTTTAATGACC | NdeI |
| KT31 | 5‘‑TTTGGATCCTCTCTGTACGCCTCCCTCAAAATAC | BamHI |
| **Screening oligos** | |  |
| ***mpn244*** | |  |
| KT17 | 5‘‑CTCCAGAGCGTTTTACTGACACT |  |
| KT18 | 5‘‑GTATGGCATTATTCCCGGCATTG |  |
| ***mpn140*** | |  |
| KT63 | 5‘‑AGTCTACAGTTATTTAGGCGGATCG |  |
| KT64 | 5‘‑CCTAAAGACACCTATACTCAAAGGC |  |
| ***mpn549*** | |  |
| KT19 | 5‘‑GAGTTGAGTCATATTAGTCTGGAAGG |  |
| KT20 | 5‘‑GCAAACCGTGGTATGAAGCTAAG |  |
| KT65 | 5‘‑CAGGTATTACGATCAATTCGGTGC |  |
| KT66 | 5‘‑GTGGCATAGACAGTCCAGTAGC |  |
| **Tn4001** | |  |
| SH29 | 5‘‑ATGAGTGAGCTAACTCACAG |  |
| SH30 | 5‘‑CAATACGCAAACCGCCTC |  |

Restriction sites are underlined, mutation sites in bold letters, p denotes phosphorylation
